# Treatment of Kidney Stones Using Extracorporeal Shock Wave Lithotripsy (ESWL) and Double-J Stent in Infants

**DOI:** 10.1155/2012/589038

**Published:** 2012-04-09

**Authors:** Mehdi Younesi Rostami, Mehrdad Taghipour-Gorgikolai, Rayka Sharifian

**Affiliations:** ^1^Department of Urology, Faculty of Medicine, Mazandaran University of Medical Sciences, Sari 4813894393, Iran; ^2^Student Research Committee, Cancer Research Center, Thalassemia Research Center, Faculty of Medicine, Mazandaran University of Medical Sciences, Sari 4813894393, Iran

## Abstract

*Background*. Extracorporeal shock wave lithotripsy (ESWL) has progressively acquired popularity as being the gold standard treatment for upper urinary tract lithiasis in infants since 1980. Our aim was to evaluate the outcome of ESWL for kidney stones and the use of double-J stent in infants. *Material and Methods*. A prospective clinical trial study performed on 50 infants with renal calculi at pelvic admitted in the Urology ward of Shafa Hospital, Sari, Iran, between 2001 and 2010. Main outcome measure of our study was clearing stones after one or more consecutive sessions of ESWL. *Results*. The study included 50 patients with renal calculi at pelvic. Among them, there were 35 (70%) boys and 15 (30%) girls with the age ranging from 1 to 13 months (mean of 7 month ± 3 days). All of them were treated by standard ESWL using Simons Lithostor plus machine. The stone sizes ranged from 6 mm to 22 mm. Double-J stents were placed in 11 infants (22%) with stones larger than 13 mm. Most of the patients required only one ESWL session. *Conclusion*. Since there were no complications following ESWL treatment, we can conclude that, in short term, ESWL is an effective and safe treatment modality for renal lithiasis in infants. In addition, we recommend double-J stent in infants with stones larger than 13 mm.

## 1. Introduction

For a long period of time, stone treatment in some patients has been a matter of controversy for urologists. Complex stones were traditionally removed by surgical intervention [1]. However, the surgical management of urolithiasis has now largely been replaced with a minimal invasive procedure-like extracorporeal shock wave lithotripsy (ESWL) [2]. The introduction of ESWL in 1980 revolutionized urolithiasis treatment [3], and now it is accepted as a highly efficacious treatment modality for most renal calculi in the pediatric population [4]. Preoperative and long-term follow-up in the pediatric population treated with ESWL suggest is minimal deleterious effects on functional measures, overall growth, and development of kidneys [5, 6]. Perhaps the good results are due to the better shock wave transmission through the smaller body volume in infants in comparison with that of the adults [7].

Although evidence has accumulated on the efficacy of ESWL in treating calculi in infants [8–10], the effect of shock wave on the pediatric urinary tract still needs to be clarified. In this study we evaluated the efficacy of ESWL in infants with renal stones, with regard to the ability of ureters to transport the fragments and the need for adjunctive procedures such as double-J stent.

## 2. Material and Method

50 infant patients with renal calculi at pelvic admitted in the Urology ward of Shafa Hospital, an educational hospital under the supervision of Mazandaran University of Medical Sciences in Sari, Iran, between 2001 and 2010, were enrolled in this study. This study was performed with the aim of evaluating and assessing the outcome of ESWL for kidney stones and the use of double-J stent in infants.

Patients with urinary tract infection and fever and chills were initially treated with antibiotics to have their infection controlled and then entered the study. Half an hour before ESWL, midazolam 0.5 mg/Kg mixed with milk and water was administered to the patients for pain relief and analgesia. All patients received Lasix 2-3 mg approximately half an hour before the operation to accumulate the urine in proximal to stone. Urine accumulation could help to clarify the stones under the guide of ultrasound to be the center of focus and be knocked down more carefully. The process would be repeated in second session in the case that the patients did not respond to ESWL the first time. Then the results of ESWL were examined with sonography, KUB, history of stone expulsion, and the symptoms after two weeks. If pieces of stones remained, patients were followed up for eight months. Urine culture, urinalysis, BUN and serum creatinine, sonography, and intravenous ureterography (IVU) would be evaluated if needed. ESWL was done under the guide of sonography with Simons Lithostor Plus machine. One thousand to 1500 shockwaves with the intensity of 2 and 3 were placed into the stones, and then KUB and ultrasound were performed again within two weeks after the treatment. After six months, ultrasound was repeated to determine the size of kidneys, remained stones and the effect of ESWL on the growth of threatened kidneys. Also urinalysis, BUN, and creatinine were tested to evaluate the function of kidneys before and after ESWL.

The obtained data were fed in SPSS version 18.0, and statistical analyses were done using *χ* ^2^ test and *t*-test (*P* < 0.05). 

## 3. Result

All the 50 patients treated on ESWL from 2001 to 2010 were infants between 31 days and 13 months. The average number of shock waves administered per session was 1500, and the intensity was grossly adjusted for the infant size and weight. The stone sizes ranged from 6 mm to 22 mm in the largest diameter, and double-J stents were replaced in 11 infants (22%) with stones larger than 13 mm.

Among them, 35 (70%) were boys and 15 (30%) were girls (M.F: 2.3 : 1) with the mean age of seven months (ranging from 31 days to 13 months). In 28 (56%) infants the stones were in the right side, in 20 (40%) in the left side, and in 2 (4%) renal stones were in both sides. All the renal stones were treated under ultrasound guidance, while both ureteral stones were treated under fluoroscopic guidance. In this study 39 (78%) infants cleared their stones in one session, nine (18%) required two sessions, and two (4%) infants required three sessions. Later, all these infants passed their fragments spontaneously, and the stones were analyzed after expulsion (Table 1). At the end of the study, all of infants were stone-free. Uncontrolled complications were not encountered after ESWL in any of patients, two (4%) infants developed fever, and they were conservatively treated with analgesics and antibiotics. Mild hematuria was seen in all infants for 24 to 48 hours which was subsided with conservative management. None of our patients developed renal hematoma or bruises on the treatment side. After 48 hours, patients were able to resume their normal activities, and none of them required open lithotripsy.

## 4. Discussion

Urolithiasis in childhood is rare compared to that of the adults and comprises 1.3% to 2% of the population with stone in the urinary system [11], and ESWL is the first option for the treatment of most renal or ureteric stones [12, 13]. Endemicity of stone diseases in infants and its recurring nature causing renal damage and end stage renal failure makes a strong case for the application of minimally invasive or noninvasive methods of treatment instead of repeated open surgery [14].

In 1989, Nijman et al. reported a success rate of 79% at 6 months after ESWL in a series of 73 infants. Myers et al., in a multicenter study working on 446 infants treated for renal (238 cases) or ureteric (208 cases) stones, reported a 68% success rate after one session and 78% after several sessions (with multiple sessions used in 35 ± 7% of the patients, depending on the treating centre) [15, 16]. In this paper, ESWL was successful in 39 (78%) patients after one session and in 9 (18%) after two sessions, and in 2 (4%) after three sessions confirming that this technique is efficient in infants similar to that of our previous series of older infants. There were also concerns whether thin pediatric ureter is capable to transport stone fragments after ESWL as efficiently as the adult ureter is. Gofrit et al. and Muslumanoglu et al. agree that pediatric ureter is more capable than adults' in transporting the stone fragments after ESWL, because infants are more active than adults, and this is known to favor stone passage [17, 18].

The use of ureteral double-J stent prior to extracorporeal shock wave lithotripsy is controversial. Most urologists prefer to use a stent in shock wave lithotripsy procedure for stones larger than 20 mm, to prevent the risk of developing steinstrasse. Sulaiman et al. found that the incidence of steinstrasse was 6.3% [19]. When the stone is less than 10 mm, stents are only used occasionally. For stones between 10 mm and 20 mm there appears to be no general consensus about the usefulness of stenting [20]. In this study we used DJ stent for stones between 13 mm and 22 mm in 11 patients, and it seems that stent prevents the developing steinstrasse.

Similar to Brinkmann et al. [21] and Elsobky et al. [22], we encountered minor complications during this study, and no case of renal hematoma or bruises on the therapy side was observed in our series. It seems that these results are due to the advances in the localization of lithotripsy field and greater concentration of import power center. All patients had gross hematuria, and findings suggest that ESWL-induced hematurias usually result from damage to renal tissue rather than from movement of calculus particles through the urinary tract [23]. But after eight-month follow-up, none of the patients had a problem.

## 5. Conclusion

Shock wave lithotripsy has been considered a mainstay of therapy in renal calculi for the last 20 years. ESWL is a noninvasive method and requires the least anesthesia among the treatment modalities. In the last decade, however, there have been changes in thinking regarding methods of patient selection for ESWL and the technique of the existing ESWL, especially, for complex patients. In this study, the results showed that after several treatment sessions in infants, ESWL did not appear to be harmful to the renal parenchyma and had no other significant complications developed during the eight months of followup. Also patients were able to resume their normal activities the day after treatment.

An important point that should always be considered is that lithotripsy by extracorporeal method can only treat available stones and does not have any effects on their recurrence. With this prospective clinical trial, we found that in infants with large calices stones (13–22 mm) it is better to use double-J stent 3 weeks before ESWL.

## Figures and Tables

**Table 1:** Clinical, demographical, and stone analysis data of patients with urolithiasis.

Characteristics	Statistics
Patients	50
Male	35 (70%)
Female	15 (30%)
Mean age at diagnosis, months	7 months ± 3 days
Stone size	
<13 mm	39 (78%)
13–22 mm	11 (22%)
Stone location	
Right urinary system	28 (56%)
Left urinary system	20 (40%)
Mixed sites	2 (4%)
Analyze of stone	
Calcium oxalate	80%
Calcium phosphate	16%
Uric acid	4%
